# Effects of general anesthesia combined with ultrasound-guided lumbosacral plexus block on hemodynamics and postoperative rehabilitation in geriatric patients with total hip arthroplasty

**DOI:** 10.4314/ahs.v24i4.38

**Published:** 2024-12

**Authors:** Yanan Cui, Qin Fan, Na Wu, Baichun Xing

**Affiliations:** Department of anesthesiology, Heping Hospital Affilicated to Changzhi Medical College, Changzhi, Shanxi, 046000

**Keywords:** Geriatric, total hip arthroplasty, general anesthesia, ultrasound guidance, lumbosacral plexus block

## Abstract

**Background:**

The incidence of fractures increases with age, and it is estimated that the incidence in women is higher than that in men.

**Objective:**

To investigate the influence of general anesthesia & ultrasound-guided lumbosacral plexus block in elderly patients with total hip arthroplasty (THA).

**Methods:**

Eighty-one geriatric sufferers who were sent to our hospital from July 2019 to January 2021 and underwent THA surgery in the Department of Orthopedics were divided into Grades I to III according to American Society of Anesthesiologists (ASA). Forty-one patients were ascribed to a combination group (using general anesthesia combined with guided lumbosacral plexus block), and forty to a general anesthesia group (with general anesthesia) by a random number table to compare the differences in mean arterial pressure (MAP), heart rate (HR), pulse oxygen saturation (SpO2), intraoperative general anesthesia dosage, incidence of postoperative delirium (POD), serum inflammation and oxidative stress indexes between them.

**Results:**

Prior to anesthesia induction, MAP, HR, and SpO2 between them had significant differences (P>0.05); at skin-incision-time and implantation of the prosthesis, the MAP value of the experimental group was higher than that of the general anesthesia group, 30 minutes after surgery, the SpO2 was higher than the general anesthesia group, and had significant differences (P<0.05); prior to the surgery, had significant differences in measured values such as Cor, blood glucose, IL-6, CRP, MDA and SOD between the two groups (P>0.05); 2 hours after that, the values of Cor, CRP, and MDA in experimental group were lower than the general anesthesia group, and that of IL-6 and SOD were higher than those in the general anesthesia group, and had significant differences (P<0.05); the dosage of propofol, remifentanil, sufentanil in experimental group were lower than the general anesthesia group, while postoperative extubation time in experimental group was earlier than general anesthesia group, and had significant differences (P<0.05); the incidence of POD in experimental group was lower than the general anesthesia group at 1d, 2d, and 3d after surgery, and had significant differences (P<0.05); the incidence of adverse reactions of anesthesia in experimental group was lower than the general anesthesia group with 9.76 % to 27.50 %, and had significant differences (P<0.05).

**Conclusion:**

General anesthesia & ultrasound-guided lumbosacral plexus block in THA patients is more conducive to the patients' hemodynamic stability, reducing inflammatory stress response, the dosage of anesthetic drugs, and the incidence of POD, compared with general anesthesia alone.

## Introduction

The elderly are prone to fall and cause femoral neck fractures due to physiological osteoporosis and weakened resilience. Conservative treatment can cause complications such as hypostatic pneumonia, pressure ulcers, and lower extremity venous thrombosis, and long-term lack of exercise can easily lead to less bone mass, resulting in non-union or poor union of fracture ends 1. Total hip arthroplasty (THA), a common method for clinic to treat femoral neck fractures in the geriatric, can quickly restore the patients' ability to get out of bed and improve the patients' life by installing a human joint prosthesis 2.

General anesthesia is currently the preferred anesthesia method for THA surgery, but the drugs cannot completely suppress the surgical stress, while blindly increasing the dose of drugs will increase the risk of adverse reactions, and even cause postoperative delirium (POD) and irreversible cognitive function obstacle 3. With the development of ultrasound technology, ultrasound-guided nerve block anesthesia has been applied in geriatric patients, which has certain advantages in reducing the dosage of general anesthesia and adverse reactions of anesthesia^4^. However, the ultrasound-guided nerve block anesthesia has not been widely used in the elderly replacement of the total hip joint, and its effects on this surgery are not clear. This study investigated the anesthesia effect of general anesthesia combined with ultrasound-guided lumbosacral plexus block in geriatric patients with THA, and observed its effect on the degree of patients' stress response. The report is as follows.

**Core tips:** THA surgery, the main treatment for geriatric patients with femoral neck fracture, of which anesthesia method can influence patients' prognosis. Existing clinical researches have confirmed general anesthesia combined with ultrasound-guided lumbosacral plexus block has better anesthesia effect. This study aims to explore the advantages of general anesthesia combined with ultrasound-guided lumbosacral plexus block based on hemodynamic indicators, inflammatory indicators and oxidative stress indicators. It found that the combined anesthesia scheme can effectively maintain the stability of perioperative hemodynamic indicators, reduce inflammation and oxidative stress, which is an important mechanism for maintaining perioperative safety and reducing POD in geriatric THA person.

## Materials and methods

### General information

Eighty-one geriatric who were sent to our hospital from July 2019 to January 2021 and underwent THA surgery in the Department of Orthopedics were described as two groups. Forty-one of them were ascribed to combination group (using general anesthesia combined with guided lumbosacral plexus block), and forty to a general anesthesia group (with general anesthesia) by a random number table.

**Inclusion criteria:** (1) The age of the included subjects was 65-78 years old; (2) The patients suffered from unilateral femoral neck fracture due to trauma and accepted THA surgery; (3) ASA grading range I to III; (4) The research programme met the requirements of medical ethics expert group in our hosital, and signed informed consent with patients and their families before treatment.

**Exclusion criteria:** (1) patients with a history of infection; (2) hematological diseases (blood tumors, severe anemia, coagulation dysfunction); (3) patients with mental illness or dementia; (4) fractures caused by tumours, tuberculosis and other diseases (5) abnormal thyroid function; (6) endocrine function disease; (7) A history of drug addiction and opioid addiction.

### Methods of anesthesia

Patients in general anesthesia one had surgery with general anesthesia. After entering the operating room, they were given ECG monitoring and oxygen inhalation masks. Anesthesia induction: propofol 1.0 mg/kg, sufentanil 0.1 µg/kg, and rocuronium 0.6 mg/kg were intravenously injected sequentially. Then, tracheal intubation would be performed for the connection with an anesthesia machine to control breathing, and maintained intraoperative PETCO2 level of 35-45 mmHg and SPO2>95 %. Micropump infusion of propofol 2-5 mg/(kg·h), remifentanil 0.04-0.4 µg/(kg·min), and rocuronium bromide muscle relaxation were added later. During the surgery, the BIS value should be maintained at 40-60, and blood pressure and HR should not fluctuate more than 20 % of the base value. Rocuronium bromide was stopped half an hour before finishing surgery, while propofol and remifentanil were stopped at that time.

In experimental one, general anesthesia & ultrasound-guided lumbosacral plexus block was used. With the supine position, the high-frequency ultrasound probe was placed at inguinal ligament of the patient to find the femoral nerve. Then, the needle was inserted into the lateral sheath of the femoral nerve and injected into the head with 0.25 % ropivacaine for 25 to 35 mL. The operation was successful when the pharmaceutical diffused in the sheath. 5 minutes later, the patient should change to the decubitus position on the unaffected side, with the affected side on top. The low-frequency ultrasound probe was placed at the mid-medial half of the line between greater trochanter and spina iliaca posterior superior with it sliding inward and downward, and 20 mL of 0.25 % ropivacaine was injected above the ilium on the lower side of the foramen magnum. Then backed to the supine position with a mask to have a preoxygenation, and performed general anesthesia with the same method as that of the general anesthesia group.

If the patient had blood pressure fluctuation, respiratory depression and other anesthetic adverse effects during the operation, vasoactive drugs and respiratory stimulants would be used, and the ventilator parameters would be adjusted.

### Observation indicators and detection methods

Compared mean arterial pressure (MAP), heart rate (HR), and pulse oxygen saturation (SpO2) of the two parts before anesthesia induction, during skin incision, implant placement, and the finish time; the operative time, intraoperative blood loss, intraoperative fluid replacement volume, propofol dosage, remifentanil dosage, sufentanil dosage, postoperative extubation time, postoperative time out of bed and discharge time; incidence of postoperative delirium (POD); serum cortisol (Cor), blood glucose, interleukin-6 (IL-6), C-reactive protein (CRP), malondialdehyde (MDA), superoxide dismutase (SOD).

Postoperative delirium (POD) before and after surgery was assessed by the Confusion Assessment Scale (CAM), which was used to assess the incidence of delirium in patients, a: The patient has acute onset or mental fluctuation; b: The patient has obvious concentration disorder; c: The patient has obvious confusion of thinking; d: Typical manifestations of abnormal conscious state of patients; clinicians are more aware of the actual situation of the patient, if prerequisites a and b are satisfied, any appearance of c or d can be judged as delirium^5^.

Before and 2 hours after that, 5 ml peripheral venous blood samples should be drawn from patients for centrifugation (parameters: 3000 r/min, 10 min) to get the serum to detect fasting blood glucose (glucose oxidase method). Detective instruments: Biochemical Analyzer (Japan Hitachi, Model: 7600). Detection of serum Cor, IL-6, CRP (enzyme-linked immunosorbent assay), Cor, IL-6, CRP kit (Shanghai Enzyme Link Biotechnology Co., Ltd.); Detection of MDA (thiobarbituric acid colorimetric method), detection of SOD (xanthine oxidase method), MDA, SOD kits (Nanjing Jiancheng Bioengineering Institute). Detective instruments: microplate reader (Shenzhen Mindray Company, model: RT-96A).

### Statistical processing

Normal distribution test showed that Cor, blood glucose, IL-6, CRP, MDA, SOD and other measurement indexes of patients in this study conformed to approximate normal distribution or normal distribution, expressed as (±s), and the comparison between them were made by t-test; χ2 test was for non-ranked count data between them; professional SPSS21.0 software was for data handling, and the test level was α=0.05.

## Results

### Comparison of baseline data between two groups

There was no significant difference in age, BMI, gender, smoking, drinking, ASA grading, Ipsilateral distribution, trauma cause, and concomitant disease between the experimental one and the control one (P>0.05). These subjects were well comparable and balanced; Table 1.

**Table 1:** Comparison of baseline data of two groups

Baseline data	Experimental group (n=41)	General anesthesia one (n=40)	t/X^2^	*P*
Age (years)	72.6±3.9	71.8±4.3	0.877	0.383
BMI (kg/m^2^)	23.82±1.70	24.06±1.84	-0.610	0.544
Male/Female	24/17	20/20	0.595	0.441
Life history(%)			0.767	0.381
Smoking	15(36.59)	11(27.50)		
Drinking	13(31.71)	9(22.50)		
Cause of trauma(%)			3.047	0.384
Car accident	13(31.71)	17(42.50)		
Fall	16(39.02)	11(27.50)		
Hit by heavy objects	6(14.63)	9(22.50)		
Other	6(14.63)	3(7.50)		
ASA Grading(%)			2.512	0.285
I grade	6(14.63)	10(25.00)		
II grade	20(48.78)	21(52.50)		
III grade	15(36.59)	9(22.50)		
Ipsilateral distribution (%)			1.010	0.315
left	22(53.66)	17(42.50)		
Right	19(46.34)	23(57.50)		
Concomitant disease(%)				
Hypertension	27(65.85)	22(55.00)	0.998	0.318
Diabetes	11(26.83)	15(37.50)	0.901	0.342
Coronary heart disease	5(12.2)	2(5.00)	1.328	0.249
Dyslipidemia	25(60.98)	19(47.50)	1.482	0.224

### Comparison of fluctuations in MAP, HR and SpO2 values between two groups

Before anesthesia induction, MAP, HR, and SpO2 between the two groups had no significant differences(P>0.05); at skin-incision-time and prosthesis-implantation, the MAP value in experimental group was higher than that of the general anesthesia group, 30 minutes after the surgery, the SpO2 value was higher than that of the general anesthesia group, and the differences were statistically significant (P<0.05); Table 2.

**Table 2:** Comparison of fluctuations in MAP, HR and SpO2 values between two groups (±s)

Index	Group	Before anesthesia induction	When cutting the skin	Implant prosthesis	30min after surgery
MAP(mmHg)	Experimental group (n=41)	96.34±4.81	85.64±3.50	83.60±3.94	98.71±4.70
	General anesthesia group(n=40)	98.03±4.57	83.91±4.00	81.51±4.28	99.04±4.93
	t	-1.620	2.073	2.287	-0.308
	*P*	0.109	0.041	0.025	0.759
HR (次/min)	Experimental group (n=41)	79.63±5.02	72.34±5.70	70.03±6.14	83.05±6.28
	General anesthesia group(n=40)	81.40±4.96	70.88±6.21	68.95±6.80	85.12±7.32
	t	-1.596	1.103	0.751	-1.367
	*P*	0.114	0.273	0.455	0.175
SpO2(%)	Experimental group (n=41)	98.26±0.64	97.13±0.50	97.24±0.39	97.95±0.40
	General anesthesia group(n=40)	98.10±0.58	96.98±0.48	97.08±0.43	97.76±0.38
	t	1.178	1.377	1.755	2.191
	*P*	0.242	0.172	0.083	0.031

### Comparison of changes in inflammation and stress indicators between two groups

Prior to the surgery, the measured values such as Cor, blood glucose, IL-6, CRP, MDA and SOD between the two groups had no significant differences (P>0.05); 2 hours after surgery, the values of Cor, CRP, and MDA in experimental group were lower than those in the general anesthesia group, and that of IL-6 and SOD were higher than those in the general anesthesia group, and the differences were statistically significant (P<0.05); Table 3.

**Table 3:** Comparison of changes in inflammation and stress indexes between two groups (±s)

Group	n	Cor (mg/L)		Blood sugar (mmol/L)		IL-6 (pg/mL)	

		Preoperative	2 hours after surgery	Preoperative	2 hours after surgery	2 hours after surgery	Preoperative
Experimental group	41	218.4±25.4	295.1±30.5	5.61±0.72	6.11±0.67	78.51±11.65	62.94±9.54
General anesthesia group	40	222.1±27.0	340.3±37.6	5.48±0.68	6.24±0.73	80.66±13.01	55.81±10.30
t		-0.635	-5.949	0.835	-0.835	-0.784	3.233
*P*		0.527	0.000	0.406	0.406	0.435	0.002



Group	n	CRP (mg/L)		MDA (nmol/mL)		SOD (U/mL)	

		Preoperative	2 hours after surgery	2 hours after surgery	Preoperative	Preoperative	2 hours after surgery

Experimental group	41	8.42±1.96	16.12±3.30	6.93±1.60	9.57±2.21	67.84±10.40	58.20±9.55
General anesthesia group	40	8.84±2.00	18.40±3.57	6.56±1.73	11.43±2.84	69.76±12.45	53.48±10.03
t		-0.955	-2.986	1.000	-3.294	-0.754	2.169
*P*		0.343	0.004	0.321	0.001	0.453	0.033

### Comparison of surgical conditions and the dosage of anesthetics between the two groups

The dosage of propofol, remifentanil, and sufentanil in experimental group were lower than those of the general anesthesia group, while postoperative extubation time in experimental group was earlier than that of the general anesthesia group, and the differences were statistically significant (P<0.05); Table 4.

**Table 4:** Comparison of surgical conditions and dosage of anesthetics between two groups

Group	n	surgery time (min)	Intraoperative blood loss (mL)	Intraoperative fluid volume (mL)	Propofol dosage (mg)	Remifentanil dosage (µg)	Sufentanil dosage (µg)	Postoperative extubation time (min)	Post-operative landing time (d)	Discharge time (d)
Experimental group	41	84.81±9.40	438.1±67.4	1366.2±184.5	244.1±30.6	398.1±57.4	9.43±2.01	5.61±1.10	2.51±0.66	7.51±1.43
General anesthesia group	40	86.10±9.52	446.3±72.5	1383.7±196.2	318.4±43.7	488.6±72.0	16.81±2.93	7.32±1.48	2.67±0.62	7.94±1.56
t		-0.614	-0.527	-0.414	-8.882	-6.263	-13.247	-5.912	-1.124	-1.294
*P*		0.541	0.599	0.680	0.000	0.000	0.000	0.000	0.264	0.200

### Comparison of the incidence of POD between patients after surgery

The incidence of POD in experimental group was lower than that of the general anesthesia group at 1d, 2d, and 3d after surgery, and the differences were statistically significant (P<0.05); Table 5.

**Table 5:** Comparison of the incidence of POD between two groups after surgery[n (%)]

Group	n	1d after surgery	2d after surgery	3d after surgery	4d after surgery
Experimental group	41	3(7.32)	2(4.88)	1(2.44)	0(0.00)
General anesthesia group	40	11(27.50)	9(22.50)	7(17.50)	2(5.00)
χ^2^		5.769	5.357	5.160	2.102
*P*		0.016	0.021	0.023	0.147

### Comparison of adverse reactions of anesthesia between two groups

The incidence of adverse reactions of anesthesia in experimental one was lower than the general anesthesia one with 9.76 % to 27.50 %, and had significant differences (P<0.05); Table 6.

**Table 6:** Comparison of adverse reactions of anesthesia between two groups

Group	n	Low blood pressure	Respiratory depression	Malignant	Tachycardia	Adverse reaction (%)
Experimental group	41	0	1	3	0	4(9.76)
General anesthesia group	40	2	3	6	1	11(27.50)
χ^2^						4.225
*P*						0.040

## Discussion

The elderly is a high-risk group of femoral neck fractures. THA is the preferred surgical method for elderly femoral neck fractures as a large number of studies have confirmed that THA is better than conservative treatment in improving the prognosis of geriatric patients 6. However, the elderly often has a variety of chronic diseases and their vital organ reserve function is reduced, so their adaptability to surgical stress is poor 7. Anesthesia is the key to ensure the smooth implementation of the surgery. Due to the lumbar bone hyperplasia and calcification in the elderly, the puncture is so difficult that the application of spinal anesthesia is limited 8. General anesthesia is widely used in elderly lower extremity surgery and can maintain hemodynamic stability during the surgery. However, the drugs can cause a variety of adverse reactions and adversely affect the prognosis of patients 9.

With the development of ultrasound technology, more and more applications have been used in anesthesia. Ultrasound-guided nerve block anesthesia has the advantages of accurate localization and little impact on hemodynamics, which is pleased to be used in the clinic 10. Sacral plexus block can affect most of the nerves in the hip to relieve pain, and block the obturator nerve in a certain extent 11. The study found that the dosage of propofol, remifentanil and sufentanil in experimental group was lower than those of the general anesthesia group, while postoperative extubation time in experimental group was shorter than that in the general anesthesia group. The aforesaid results suggest that general anesthesia&ultrasound-guided lumbosacral plexus block in THA patients is more conducive to reducing the dosage of general anesthesia drugs and promoting postoperative extubation than general anesthesia alone for the lumbar plexus and sacral plexus innervating the sensations in all directions of the thigh and buttocks, and lumbosacral plexus block basically meeting the needs of THA surgery, so that general anesthesia can be implemented with a smaller dose of drugs to get good analgesic and sedative effects. The patients' body can also suffer from less stress and recover faster after the surgery 12-13.

Under the stimulation of surgical trauma, it will inevitably cause a stress response, resulting in an increase in hemodynamic indicators such as MAP and heart rate 14. Respiratory depression caused by general anesthesia drugs can lead to hypoxia and decrease SpO2 levels 15. When the body is under stress, blood glucose levels rise, stress hormones such as Cor are secreted in large quantities, inflammatory factors such as IL-6 and CRP, and oxygen free radicals are produced in large quantities, resulting in lipid peroxidation damage to biological membranes, and a large amount of by-product MDA is generated 16. SOD, an auto antioxidant enzyme, will consume in large quantities when oxidative stress occurs, resulting in decreased activity 17. The study found that the MAP values of the experimental group at the time of skin incision and implant placement were higher than those of the general anesthesia group, while SpO2 value at 30 minutes after the surgery was higher than that of the general anesthesia group; 2h after surgery, the measured values of Cor, CRP and MDA in experimental group were lower than those of the general anesthesia group, and the measured values of IL-6 and SOD were higher than those of the general anesthesia group. The aforesaid suggest that combined application of general anesthesia and ultrasound-guided lumbosacral plexus block in THA patients is more conducive to hemodynamic stability and lessening inflammatory stress response than general anesthesia alone. As it has been confirmed by researches that the combined application of ultrasound-guided lumbosacral plexus block and general anesthesia strengthening the effect of anesthesia, and making up for the poor effect of nerve block in the surgical area to block the conduction of peripheral noxious stimulation signals to the central nervous system better, thus producing pre-emptive analgesia to reduce the secretion of stress hormones caused by surgical trauma, which is conducive to maintaining intraoperative hemodynamic stability 18.

POD refers to acute and volatile brain dysfunction in patients after surgery, with advanced age as an independent risk. POD is not only severe to cause irreversible cognitive impairment, but also may increase the risk of postoperative mortality 19. The research found that the incidence of POD in experimental one was lower than the general anesthesia one at 1d, 2d, and 3d after the surgery, and the incidence of adverse reactions of anesthesia was lower than the general anesthesia one, which suggested that the combined application of general anesthesia and ultrasound-guided lumbosacral plexus block in THA patients is more conducive to reducing adverse reactions and POD than general anesthesia alone. This is related to the fact that ultrasound-guided lumbosacral plexus block can reduce the dosage of general anesthesia drugs and the abnormal circulatory function and central nervous system depression caused by excessive use of general anesthesia drugs. The results of our study are consistent with those of previous studies 20.

## Conclusion

The combined application of general anesthesia and ultrasound-guided lumbosacral plexus block in THA patients is more conducive to keeping the hemodynamic in order, and reducing inflammatory stress response, the dosage of anesthetics, and the occurrence of POD than general anesthesia alone.
